# The Relationships between Physical Activity, Screen Time and Sleep Time According to the Adolescents’ Sex and the Day of the Week

**DOI:** 10.3390/healthcare10101955

**Published:** 2022-10-06

**Authors:** Daniel Sanz-Martín, José Luis Ubago-Jiménez, Germán Ruiz-Tendero, Félix Zurita-Ortega, Eduardo Melguizo-Ibáñez, Pilar Puertas-Molero

**Affiliations:** 1 Faculty of Humanities and Social Sciences, Isabel I University, 09003 Burgos, Spain; 2 Department of Didactics Musical, Plastic and Corporal Expression, Faculty of Education Science, University of Granada, 18071 Granada, Spain; 3 Department of Languages, Arts and Physical Education Teaching, Faculty of Education, Complutense University of Madrid, 28040 Madrid, Spain

**Keywords:** physical activity, screen time, sleep time, adolescents, sex, weekday, weekend

## Abstract

This study had two aims: (1) to identify the levels of physical activity, screen time and sleep time of adolescents according to the sex of the participant and the day of the week; (2) to find out the relationships between physical activity, screen time and sleep time according to the sex of the participant and the day of the week. The study design was non-experimental, descriptive-correlational and cross-sectional. The sample consisted of 694 adolescents in Compulsory Secondary Education from Soria (Spain). Four by One-Day Physical Activity Questionnaires were used to measure levels of physical activity, screen time and sleep time. Levene’s test and Student’s *t*-test were used to calculate the difference between the means of the variables. Pearson’s test was used to calculate bivariate correlations between variables. Results showed higher levels of screen time in males (136.93 min/day ± 81.548). Screen time, sleep time and moderate–vigorous physical activity are higher during the weekend. Positive relationships were found between screen time and light physical activity (*r*_males_ = 0.274; *p* ≤ 0.01; *r*_females_ = 0.065; *p* > 0.05). The correlations between moderate–vigorous physical activity and screen time were negative (*r*_males_ = −0.282; *p* ≤ 0.01; *r*_females_ = −0.187; *p* ≤ 0.05). The relationship between screen time and sleep time was negative in males (*r* = −0.135; *p* ≤ 0.05). In conclusion, the levels of physical activity, screen time and sleep time vary according to the sex of the participants and the day of the week.

## 1. Introduction

Preventable premature death represents 15% of annual global deaths, equivalent to 3.9 million deaths [1]. Physical activity has been shown to be one of the factors that contribute to the maintenance of health and the prevention of premature death [1,2]. For example, the practice of physical activity by young people improves cardiometabolic health and bone mass, reduces the risk of experiencing depression and helps in the maintenance of a healthy physical fitness and weight [3,4]. For physical activity to be beneficial, it should be performed according to World Health Organization guidelines [3]. For children and adolescents, these recommendations are 60 min/day of moderate–vigorous physical activity, which should be mostly aerobic, and should include vigorous, muscle-strengthening and bone-strengthening activities at least three days a week [3].

Despite the health concerns and evidence on how it can be avoided, global levels of physical activity are still alarming. Based on the study of Guthold et al. [5], more than 80% of adolescents do not meet physical activity practice recommendations. Knowing the physical activity levels of each particular population and the factors that influence adolescents’ movement behaviors may help to design more effective priority proposals [6,7].

Craggs et al. [8] found that sex, region of residence, living in a rural/urban area, weekend days and sedentary behavior are some of the correlational factors that most influence the physical activity levels of young people. In relation to sex, physical activity levels are higher in boys than girls. Guthold et al. [5] showed that 84.4% of females and 78.4% of males aged 11–17 years old worldwide did not meet the moderate–vigorous physical activity recommendations of 60 min/day. Xu et al. [9] found that 44.7% of 16–19-year-old males in the United States met physical activity practice recommendations, compared to 20.7% of females who did. Furthermore, physical activity levels decrease with age, and there is a negative turning point around the age of 9 [10]. De Fátima et al. [11] demonstrated that girls aged 8–10 years old were more likely to show lower trajectories of moderate–vigorous physical activity two years and seven years after the first measurement. The attainment of higher levels of physical activity in males is maintained in any segment of the day [7] and day of the week [12]. Regarding the day of the week, most adolescents are inactive on school days and weekends (65.5%) and 22% are inactive on one of these weekly segments [13]. Moreover, the percentage of active children increases during weekends, as does the percentage of very inactive children [14,15].

Screen time is also influenced by gender and is predominantly higher in males. Dahlgren et al. [16] showed that 10–15-year-old males in Sweden spent an average of 124.4 min/day of screen time, compared to 105.5 min/day for females. U.S. adolescent males spent an average of 5 h/day on screen time and females 4.1 h/day [9]. Hrafnkelsdottir et al. [17] found that 15-year-old males in Reykjavik (Iceland) spent 5.6 h/day during school days and 7 h/day during weekends on screen time, whereas females spent 5 h/day and 6.4 h/day, respectively. In addition, De Fátima et al. [11] found that females were less likely to belong to trajectories with more screen time between childhood and adolescence.

Adolescent sleep patterns are quite similar between males and females [18], including sleep time [9], although females sleep longer on average [11,19]. Sleep time decreases with age, except for a slight increase after the age of 60 [20]. Depending on the day of the week, adolescents sleep longer on weekends than on school days; moreover, females sleep longer than males on school days but less on weekends [21].

The relationship between physical activity and screen time is negative [22,23,24]. It has also been shown that sedentary time on school days is higher than during weekends, although the opposite is true for moderate–vigorous physical activity time [25,26]. In addition, non-compliance with the recommended screen time (<2 h/day) of adolescents has been demonstrated [24], which is related to body weight gain, sleep problems, musculoskeletal pain and depression [27].

Ghekiere et al. [28] conducted a study with European adolescents and showed that from 2002 to 2014, the percentage of adolescents complying with the physical activity recommendations for 5, 6 and 7 days a week increased. Similarly, the average screen time and the percentage of young people exceeding the recommended 2 h/day increased. There was also an increase in the number of participants who had daily and weekly difficulties falling asleep. Note that screen time is negatively related to sleep outcomes [29]. In addition, adolescents’ sleep time is higher on weekend days than on school days [21,30].

Tambalis et al. [31] showed that low levels of sleep were related to screening time in young people aged 8–17 years. Lee et al. [32] found that moderate–vigorous physical activity time was predictive of sleep time and sleep efficiency, with negative and positive relationships, respectively.

The scientific evidence regarding adolescent physical activity, screen time and sleep levels is substantial compared to the evidence that has studied the relationships between them in a direct measurement. There is even less evidence that differentiates the relationships according to physical activity intensity with light physical activity as a secondary factor. Likewise, available information on the differences in physical activity levels during certain daily segments is even more limited [6]. Several studies published prior to this study have exposed the need to investigate the impact of different aspects of physical activity, such as its intensity, and screen time on adolescent sleep [9], as well as the importance of studying their relationships in representative samples of adolescents [33]. This need may be partially due to the fact that physical activity, screen time and sleep time are directly related to obesity [34], which is considered one of the main problems of the 21st century [3].

Based on this evidence and with the need to know the state of the question in under-studied populations, the following research objectives are proposed: (1) to identify the levels of physical activity, screen time and sleep time of adolescents in the area of Soria according to the sex of the participant and the day of the week; (2) to know the relationships between physical activity, screen time and sleep time according to the sex of the participant and the day of the week.

## 2. Materials and Methods

### 2.1. Design and Subjects

The study is located in the behavioral epidemiology paradigm [35]. The method used was non-experimental, ex post facto, cross-sectional and descriptive-correlational of physical activity, screen time and sleep time [36].

The sampling followed was non-probabilistic, by convenience. The sample consisted of participants from 17 of the 19 schools in the area. In the participating centers, a group of students was selected for each grade following the criterion of accessibility, so that all students in each center could answer the questionnaires on the same days. The number of participating students was 1089, but 395 were excluded from the final sample. The exclusion criteria were: (1) failure to respond correctly to the questionnaire during the four days it was administered, (2) if the student was considered atypical on any of the days he/she was asked about, and (3) if the participants obtained atypical or out-of-range values in the duration of physical activity practice. As a consequence, a total of 694 adolescents in compulsory secondary education participated in the study (14.06 ± 1.27 years). The sample was representative of the adolescent population of the Soria area (Spain) with a precision error of 3.3%, for a confidence level of 95%, a standard deviation of 50 and a known population of 1236 adolescents living in this area. Table 1 shows the descriptive characteristics of the sample.

### 2.2. Instruments, Variables and Procedure

The instrument used in the study was the Four by One-Day Physical Activity Questionnaire. The questionnaire was initially designed by Cale [37] in order to be used with British adolescents. It was subsequently validated by Soler et al. [38] for use with Spanish adolescents. The Cronbach’s alpha reliability is *α* = 0.832. The Four by One-Day Physical Activity Questionnaire has been used in different research studies to examine adolescents’ physical activity behaviors [14,15,39].

The Four by One-Day Physical Activity Questionnaire is administered for four days and asks about the physical activity of the previous day, except for Saturday, which is asked about on the following Monday. The questionnaire is answered on the activities carried out on a school day during which physical education was performed, on a school day during which physical education was not performed, on a Saturday and on a Sunday. The questionnaire has two formats: one for school days and another for weekend days. The school day questionnaire is segmented into two parts: morning (also differentiated into: before class, during class breaks and at lunchtime) and afternoon–night. The weekend questionnaire is segmented into three parts: morning (further differentiated into: when getting up and at lunchtime), afternoon and night.

The physical activity variable has been assessed as a computation of the several-item questionnaire, depending on the type of activity and the amount of minutes practiced. Physical activity time was calculated as the sum of partial times and was expressed as mean minutes/day of practice. Physical activity energy expenditure was calculated by multiplying the practice time by the metabolic equivalent assigned to the activity according to the questionnaire protocol. Subsequently, two physical activity categories were established: light physical activity (between 1.5 and 2.5 metabolic equivalents/hour) and moderate–vigorous physical activity (at least 3 metabolic equivalents/hour). The recreational screen time variable was assessed as the sum of several items relating to television viewing and the use of computers, video games and the Internet. Finally, the sleep time variable was calculated by subtracting the time elapsed between waking up and falling asleep from the 24 h day. In addition, the results were calculated according to the sex of the participant (differentiating between male and female) and the day of the week. In the latter, a distinction was made between weekday (Monday to Friday) and weekend (Saturday and Sunday).

Regarding the procedure followed in the investigation, a documentary search was initially carried out on evidence relating to the research topic. Subsequently, the research project was drafted and the required permissions were obtained. The research respected the principles of the Declaration of Helsinki and was approved by the Ethics Committee of the University of Granada (1478/CEIH/2020). Permission was also obtained from the head of the Provincial Directorate of Education of Soria. In addition, an informed consent form was given to the families of the selected participants. Only young people who gave permission signed by their legal guardians participated. The questionnaire was then administered in paper format and there was one interviewer for every six students, as specified in the questionnaire protocol. The results obtained were analyzed and the final report was drafted.

### 2.3. Data Analysis

IBM SPSS 26.0 software (International Business Machines Corporation, Armonk, NY, USA) was used for the statistical analysis. Initially, the data were cleaned and no missing values or outliers were found. Basic descriptive values were then calculated, such as means and frequencies. Next, the Kolmogorov–Smirnov test was applied, noting that the variables followed normal distributions.

Levene’s test and Student’s *t*-test were used to calculate the difference between the means of the variables. The first test was used to measure the homogeneity of variances. Student’s *t*-test was then calculated for independent samples, including the level of bilateral significance. This complemented the effect size using Cohen’s standardized measure d and the 95% confidence interval. The effect size was considered as either zero (0–0.19), low (0.20–0.49), moderate (0.50–0.79) or high (≥0.80) [40]. Pearson’s test was used to calculate bivariate correlations between variables.

## 3. Results

Table 2 shows the levels of screen time, sleep time and physical activity of adolescents in the area of Soria. In addition to the mean physical activity levels, we also found that the levels differentiated by sex of the participants and day of the week. Physical activity is differentiated in time of practice and energy expenditure and according to intensity (light physical activity, moderate–vigorous physical activity or average physical activity). Young people spend more time on screens on the weekend. This is also true whether or not they are males or females. Males spend more time on screens than females, regardless of the type of day. Adolescents sleep longer on the weekend than on school days. Moreover, by sex, males sleep longer on average and also on school days than females, but the opposite is true on weekends. Females obtain higher levels of light physical activity time and energy expenditure in all time–week categories. In addition, levels of moderate–vigorous physical activity in both units of measurement are higher in males. Significant differences were obtained in comparing physical activity means according to the sex of the participant. Mean physical activity time is higher in females, as well as during school days. Conversely, energy expenditure in physical activity is higher in males. In addition, the mean values are also higher during the school day than on weekends.

Table 3 shows the correlations between physical activity, screen time and sleep time variables for males and females, respectively. In males, light physical activity time and energy expenditure are positively correlated with screen time. In contrast, the dimensions of moderate–vigorous physical activity were negatively related to screen time. Mean levels of physical activity time are positively related to screen time, but the relationship is negative in regard to energy expenditure. The trend is similar when comparing these variables according to the day of the week. All of the relationships between the different physical activity variables and sleep time are negative and significant for males, with moderate intensity. The relationships between sleep time and moderate–vigorous physical activity with time and energy expenditure are negative, approximately 0 and not significant. Most of the relationships by day of the week are similar, except for sleep time and energy expenditure in physical activity during the weekend, which is positive and significant.

Table 4 shows the relationships between the variables for females. Screen time and light physical activity are positively, but not significantly, related to time and energy expenditure. Screen time and moderate–vigorous physical activity are negatively and significantly related. In addition, physical activity and screen time are negatively related and have a negative relationship with energy expenditure. Comparing these variables according to the day of the week, the same trend was observed, except for screen time–moderate–vigorous physical activity time during the school day and energy expenditure in light physical activity–screen time during the school day. The relationship between females’ physical activity and sleep time is negative, moderate and significant. There is an exception for moderate–vigorous physical activity and sleep time, and energy expenditure in moderate–vigorous physical activity, which are still negative, but close to zero and not significant. Comparing these relationships according to time categories, we find that all relationships except those linked to moderate–vigorous physical activity follow the same trend. It should be noted that moderate–vigorous physical activity time, energy expenditure and sleep time are positively related, close to 0 and not significant.

## 4. Discussion

In the present study, the levels of physical activity, screen time and sleep time of adolescents in the area of Soria were identified. The correlations between these variables were also identified. This information has been found according to the sex of the adolescents and the day of the week. Next, a discussion involving the previously existing scientific evidence will be established.

This study found that the average levels of screen time are higher on the weekend than they are during the school day. In addition, males spend significantly more time on these activities than females on both weekends and school days. Dahlgren et al. [16] also found that boys spent more screen time as a whole. Kjellenberg et al. [41] also obtained higher results during the weekend, but girls spent more time in both weekly periods. In contrast, Zhang et al. [25] showed that Chinese adolescents spent more time on screen time on school days.

In summary, the screen time levels of adolescents in Soria are lower than in other studies. This is due, to some extent, to the fact that only the items “watching television” and “using computers, video games and the Internet” were taken into consideration. The increase could have been substantial if mobile phone/Tablet use had also been asked. Across the board, screen time reaches worrying levels, especially on weekend days. These have increased over the last decades [28] and have worsened with age [23,25].

In relation to sleep time levels, the prevalence is similar to that of screen time, i.e., higher levels on weekend days and for males. In this case, a significant sex difference is only found for school days. Peiró-Velert et al. [15] found that adolescents from Valencia (Spain) slept on average almost six minutes longer than those from Soria (551 ± 54 min/day). Bandeira et al. [30] obtained longer sleep time on weekend days. Here, the mean weekly sleep time, excluding “siesta”, was lower than in Soria, with 519 min and 525 min in each Colombian population. Grant et al. [21] also found that Montana youths slept longer during the weekend (555.3 ± 84.3 min/day) than during the school day (512.8 ± 48.6 min/day). Comparing the levels of the study conducted by Grant with those of Soria, it is observed that males and females in Soria sleep longer during both weekends and school days, with the exception of females on weekdays.

In terms of physical activity levels, differences are found according to the variable measured: sex and day of the week. The amount of light physical activity time during school days is higher than on weekends, and females spend significantly more time doing light physical activity than males. These differences are similar according to the energy expenditure of light physical activity. Likewise, the average moderate–vigorous physical activity time is higher on the weekend, and males obtain significantly higher levels in the different time segments. Similar results were obtained with regard to energy expenditure in moderate–vigorous physical activity. In relation to physical activity time, levels are higher on school days. Regarding sex, males performed more physical activity on weekends and females performed more physical activity during the school day. In addition, energy expenditure in physical activity is higher during the school day, and males are higher in both time categories. Time and energy expenditure in physical activity have a similar prevalence to light physical activity, but not to moderate–vigorous physical activity, with higher levels during weekdays. This is due to the fact that light physical activity levels are much higher in quantity.

The present study follows the trend of previous studies, with boys performing more moderate–vigorous physical activity than girls [22,25,41]. Conversely, there is a discrepancy in terms of the day of the week, which is not similar to international studies. Adolescents in Soria perform more moderate–vigorous physical activity on weekend days, in contrast to the findings of Zhang et al. [25], Grant et al. [21] and Moore et al. [42].

Comparing energy expenditure levels in physical activity, the results of adolescents in Soria are also in contrast to others conducted in Spain. In Soria, adolescents obtain higher averages on weekdays, but Peiró-Velert et al. [14] and Peiró-Velert et al. [15] show higher averages on weekends. In terms of physical activity time, adolescents from Soria and North Carolina [42] obtain higher levels on weekdays.

Next, the discussion will refer to the second research objective, which deals with the correlations between screen time, sleep time and physical activity. It should be noted that no previous study has been found which has taken the joint measurement of all these relationships according to the sex of the adolescents and the day of the week into account. Therefore, this study sets a precedent. Consequently, the discussion with previous studies will be made partially.

The relationship between screen time and physical activity of adolescents in Soria does not follow a clear trend. Screen time and light physical activity are positively related and are higher in males. In contrast, screen time and moderate–vigorous physical activity are negatively related. The same occurs with energy expenditure in physical activity. In contrast, the relationship between screen time and physical activity time is positive in males and negative in females. These general trends are maintained in males during weekdays and weekends, but not in females. For example, the relationship between screen time and physical activity time for females during the school day is positive. These relationships might not have changed substantially if other types of screen activities had been included, as, for example, there is no correlation between mobile phone time and physical activity [16]. Furthermore, it should be taken into account that the relationships between screen time and physical activity vary depending on the time of the academic year [43].

The positive relationship between light physical activity and screen time may be partially due to the fact that the Four by One-Day Physical Activity Questionnaire items “television viewing” and “the use of computers, video games and the Internet”, which were considered to calculate the screen time variable, involve an energy expenditure of 1.5 metabolic equivalents/hour, so they have also been considered to calculate the light physical activity variable.

Bejarano et al. [22] also found negative relationships between moderate–vigorous physical activity time and minutes of television viewing. This was maintained in both males and females, but was non-significant. In contrast, Braig et al. [23] found positive relationships between television viewing and physical activity, but negative relationships with other types of screen activity for 13-year-old boys and girls.

Regarding the relationships between physical activity and sleep time, the prevalence is negative and significant. The relationship between sleep and light physical activity is moderate in both males and females, both in terms of time and energy expenditure. Moreover, the trend is maintained regardless of the day of the week. The relationship between sleep and moderate–vigorous physical activity is much smaller and almost zero. Moreover, the relationship between these values in females and during the school day is positive, although close to zero. The relationships between sleep time and physical activity are perfect or almost perfect. This is due to the fact that, throughout the day, one is either sleeping or doing physical activity of some intensity. In contrast, such relationships with energy expenditure in physical activity are negative, with the exception of weekend days for males, in which the relationship is positive, moderate and significant. Lee et al. [32] also showed that sleep time negatively and significantly predicted the moderate–vigorous physical activity time of high school students. Furthermore, it was positive with respect to sleep efficiency.

The difference in trends and dominance in the relationships between screen time and physical activity and between sleep and physical activity may be due to the way time and energy expenditure were calculated in this study. Time was calculated as a sum of items. In contrast, for energy expenditure, metabolic equivalents were used according to the types of intensity established in the protocol. This would corroborate what we have previously stated and prove that physical activity intensity categories have different impacts on the relationships between variables. Thus, it would be interesting to carry out other studies similar to this one using more physical activity categories, or even cut-off points in the measurement.

The relationship between screen time and sleep time in males from Soria is negative and slight. Moreover, it is significant on school days and for the weekly average. In contrast, these relationships are positive, close to zero and non-significant in females, with the exception of the school day, which is negative. In the case of females, this could be in line with Hrafnkelsdottir et al. [17], who concluded that less screen time is associated with less sleep variability.

The differences in physical activity found in this study according to the sex of the participant may be partially due to the fact that Spanish females obtain higher physical activity abandonment scores in the items referring to body image/physical/social anxiety, fatigue/laziness and obligations/lack of sleep [44]. This physical activity abandonment by females, together with the fact that they dedicate more time to studying [45], leads to an increase in the time and energy expenditure associated with light physical activity. In addition to the above, the fact that females sleep for less time means that the average daily physical activity is also higher in females.

The study conducted with adolescents in Soria has some limitations that need to be commented on. In sleep time, “siesta” time was not taken into account, only night-time rest time. Another limitation is that no questions were asked about the time spent using a mobile phone or tablet. Thirdly, there is an inherent limitation in the instrument administered. This could have been complemented with objective physical activity measurement instruments, such as pedometers or accelerometers. There is also another limitation derived from the type of research design selected. Cross-sectional design allowed us to study the relationships between physical activity, screen time and sleep time in a specific population and over a specific period of time. This implies that the results cannot be generalized to other populations and that they could vary in the population of Soria in a short period of time after the questionnaire administration. In addition, another limitation is the consequence of having carried out a descriptive-correlational study. This type of study greatly limits the ability to infer the directionality of the correlations between variables and to deduce the temporality and causality of such data. This, in turn, implies that there may be reverse causation in the relationship between variables and that it would still be possible that physical activity and sleep time are not consequences of, but rather antecedent of, screen time.

It is advisable to use the results of this study for further research on this topic. An explanatory model could be designed to take into account the relationships between the variables as a whole and establish their combination and weighted weight. This model would also help to identify the existence and influence of variables not initially considered or hidden in the measurement of the relationship between two other variables. This would make it possible to identify which determinants to prioritize in health promotion proposals with adolescents and save resources in terms of time. Further future research could extend the age of the participants by carrying out another cross-sectional study. This study, carried out with the adolescent population of Soria, only allows us to know the relationships between the variables studied at a specific time and for that population. As no similar studies have been found with other population groups, it would be interesting to know how the relationships between the variables vary according to the age of the participants, since other studies have only shown that the levels of physical activity and sleep time decrease with age and screen time increases. Other variables, such as family socioeconomic level or social support for physical activity practice, could also be included in this cross-sectional study. In addition, in relation to the last limitation of the study mentioned previously, a longitudinal and experimental study would provide insight into how the relationships vary as the ages of the participants increase and would allow us to test the causality between the variables in order to know whether it is of the direct or reverse type. Finally, proposals should be designed to improve the physical activity levels of adolescents, especially females, during both weekdays and weekends, since neither reach the physical activity practice recommendations. Similarly, they should be designed to reduce the screen time of adolescents on weekends, since they exceed the recommended limit of 2 h per day.

## 5. Conclusions

Screen time levels are higher in males and during weekends, in which the recommended 2 h/day is exceeded. Sleep time is similar in males and females, with the exception of weekdays, which is higher in males. Light physical activity levels are mainly higher in females and moderate–vigorous physical activity levels are predominantly higher in males. The same is true for weekdays and weekends, respectively.

The relationships between screen time and light physical activity are positive and slight, and are significant for males. With respect to moderate–vigorous physical activity, these relationships are negative and mostly significant. In addition, the relationships between sleep time and physical activity are mostly negative, moderate and significant. These same relationships are maintained according to the day of the week, with some exceptions, especially in females. Likewise, the relationship between screen time and sleep time is negative in males.

## Figures and Tables

**Table 1:** Descriptive characteristics of the sample.

		Sex *n* (Percentage)	
		Male	Female
Grade	1st	105 (15.13)	64 (9.22)
	2nd	97 (13.98)	82 (11.82)
	3rd	82 (11.82)	83 (11.96)
	4th	80 (11.53)	101 (14.55)
Location of school	Urban	177 (25.50)	144 (20.75)
	Rural	187 (26.95)	186 (26.80)
Type of school	Public	296 (42.65)	257 (37.03)
	Private-concert	68 (9.80)	73 (10.52)
Total		364 (52.4)	330 (47.6)

**Table 2:** Screen time, sleep time and physical activity according to the participants’ sex.

Variable		Total Sample		Male		Female		Levene Test		T Student		ES (d)	95% CI
		M	S.D.	M	S.D.	M	S.D.	F	Sig.	t	Sig. (Bilateral)		
ST (min/day)	Weekday	63.36	53.390	74.61	56.521	50.95	46.348	10.997	0.001	6.031	0.000	0.455	[15.962; 31.370]
	Weekend	172.04	116.034	199.25	124.492	142.04	97.663	11.228	0.001	6.767	0.000	0.508	[40.609; 73.810]
	Full week	117.70	76.691	136.93	81.548	96.49	64.738	11.245	0.001	7.266	0.000	0.545	[29.511; 51.364]
SlT (min/day)	Weekday	497.21	58.258	505.37	49.695	488.21	65.340	2.219	0.137	3.917	0.000	0.297	[8.562; 25.774]
	Weekend	593.70	83.954	592.44	82.171	595.09	85.891	0.485	0.486	−0.414	0.679	0.032	[−15.182; 9.892]
	Full week	545.46	57.008	548.91	53.036	541.65	90.946	2.663	0.103	1.678	0.094	0.099	[−1.235; 15.758]
LPA time (min/day)	Weekday	874.86	73.84	856.09	68.219	893.62	74.898	0.143	0.705	−6.909	0.000	0.512	[−48.202; −26.869]
	Weekend	778.23	99.163	765.78	102.207	790.68	94.133	0.899	0.343	−3.327	0.001	0.253	[−39.589; −10.204]
	Full week	825.28	70.931	810.86	71.876	839.70	66.782	1.547	0.214	−5.460	0.000	0.414	[−39.213; −18.469]
MVPA time (min/day)	Weekday	68.35	43.686	78.54	48.354	58.17	34.956	29.567	0.000	6.401	0.000	0.478	[14.119; 26.616]
	Weekend	70.01	65.610	81.85	69.293	54.23	58.037	7.983	0.005	5.711	0.000	0.430	[18.125; 37.120]
	Full week	68.71	46.409	80.77	49.534	56.64	38.507	16.007	0.000	7.135	0.000	0.540	[19.090; 32.252]
PA time (min/day)	Weekday	942.79	58.258	934.63	49.695	951.79	65.340	2.219	0.137	−3.917	0.000	0.297	[−25.774; −8.562]
	Weekend	846.30	83.954	847.55	82.172	844.91	85.981	0.485	0.486	0.414	0.679	0.031	[−9.892; 15.182]
	Full week	893.88	57.544	891.64	55.921	896.345	59.271	1.488	0.223	−1.076	0.282	0.082	[−13.292; 3.882]
LPA—EE (MET/day)	Weekday	22.93	2.231	22.36	1.936	23.56	2.365	0.000	0.985	−7.327	0.000	0.557	[−1.519; −0.877]
	Weekend	21.45	3.070	20.98	3.078	21.96	2.984	0.004	0.948	−4.240	0.000	0.323	[−1.430; −0.525]
	Full week	22.15	2.211	21.69	2.238	22.65	2.071	0.247	0.617	−5.822	0.000	0.444	[−1.278; −0.634]
MVPA—EE (MET/day)	Weekday	5.72	4.343	6.94	5.060	4.37	2.831	96.236	0.000	8.366	0.000	0.618	[1.969; 3.178]
	Weekend	6.15	6.017	7.83	6.608	4.29	4.640	40.427	0.000	8.209	0.000	0.614	[2.687; 4.378]
	Full week	5.95	4.432	7.40	4.828	4.32	3.124	62.952	0.000	9.840	0.000	0.749	[2.480; 3.682]
PA—EE (MET/day)	Weekday	28.65	3.660	29.30	4.216	27.92	2.758	59.814	0.000	5.132	0.000	0.383	[0.849; 1.902]
	Weekend	27.59	5.266	28.81	5.674	26.25	4.409	21.425	0.000	6.655	0.000	0.500	[1.801; 3.309]
	Full week	28.10	3.764	29.10	4.145	27.00	2.927	34.793	0.000	7.349	0.000	0.580	[1.565; 2.627]

Note: Effect Size (ES); Screen time (ST); Sleep Time (SlT); Light Physical Activity (LPA); Moderate–vigorous Physical Activity (MVPA); Physical Activity (PA); Energy Expenditure (EE); Metabolic Equivalents (MET).

**Table 3:** Bivariate correlation between screen time, sleep time and physical activity for males.

	1	2	3	4	5	6	7	8	9	10	11	12	13	14	15	16	17	18	19	20	21	22	23	24
1. ST wy	-	0.562 **	0.775 **	−0.149 **	−0.043	−0.103	0.271 **	0.148 **	0.231 **	−0.229 **	−0.169 **	−0.231 **	0.149 **	0.043	0.091	0.277 **	0.105 *	0.201 **	−0.257 **	−0.201 **	−0.275 **	−0.181 **	−0.177 **	−0.219 **
2. ST wd		-	0.958 **	−0.147 **	−0.078	−0.130 *	0.221 **	0.232 **	0.255 **	−0.160 **	−0.250 **	−0.264 **	0.147 **	0.078	0.092	0.197 **	0.124 **	0.160 **	−0.184 **	−0.259 **	−0.288 **	−0.130 *	−0.234 **	−0.233 **
3. ST fw			-	−0.164 **	−0.075	−0.135 *	0.262 **	0.229 **	0.274 **	−0.202 **	−0.249 **	−0.282 **	0.164 **	0.075	0.102	0.246 **	0.131 *	0.192 **	−0.229 **	−0.267 **	−0.315 **	−0.162 **	−0.240 **	−0.254 **
4. SlT wy				-	0.248 **	0.661 **	−0.706 **	−0.279 **	−0.515 **	−0.032	0.116 *	0.048	−1 **	−0.248 **	−0.618 **	−0.686 **	−0.245 **	−0.437 **	−0.003	0.158 **	0.099	−0.319 **	0.051	−0.131 *
5. SlT wd					-	0.891 **	−0.162 **	−0.738 **	−0.538 **	−0.026	−0.097	−0.077	−0.248 **	−1 **	−0.759 **	−0.149 **	−0.582 **	−0.461 **	−0.033	−0.081	−0.068	−0.108 *	0.410 **	−0.346 *
6. SlT fw						-	−0.456 **	−0.703 **	−0.657 **	−0.035	−0.021	−0.037	−0.661 **	−0.891 **	−0.878 **	−0.436 **	−0.565 **	−0.562 **	−0.027	0.011	−0.006	−0.233 **	−0.294 **	−0.329 **
7. LPA T-wy							-	0.360 **	0.713 **	−0.685 **	−0.338 **	−0.571 **	0.706 **	0.162 **	0.409 **	0.950 **	0.306 **	0.615 **	−0.637 **	−0.369 **	−0.582 **	−0.329 **	−0.264 **	−0.358 **
8. LPA T-wd								-	0.824 **	−0.221 **	−0.599 **	−0.510 **	0.279 **	0.738 **	0.606 **	0.356 **	0.839 **	0.750 **	−0.175 **	−0.531 **	−0.439 **	−0.047	−0.152 **	−0.132 *
9. LPA T-fw									-	−0.477 **	−0.579 **	−0.631 **	0.515 **	0.538 **	0.724 **	0.685 **	0.760 **	0.925 **	−0.420 **	−0.545 **	−0.583 **	−0.190 **	−0.223 **	−0.257 **
10. MVPA T-wy										-	0.358 **	0.756 **	0.032	0.026	0.059	−0.635 **	−0.180 **	−0.418 **	0.902 **	0.358 **	0.719 **	0.791 **	0.319 **	0.640 **
11. MVPA T-wd											-	0.845 **	−0.116 *	0.097	0.007	−0.348 **	−0.577 **	−0.560 **	0.298 **	0.879 **	0.728 **	0.197 **	0.711 **	0.604 **
12. MVPA T-fw												-	−0.048	0.077	0.078	−0.546 **	−0.467 **	−0.583 **	0.657 **	0.772 **	0.892 **	0.538 **	0.646 **	0.737 **
13. PA T-wy													-	0.248 **	0.618 **	0.686 **	0.245 **	0.437 **	0.003	−0.158 **	−0.099	0.319 **	0.051	0.131 *
14. PA T-wd														-	0.759 **	0.149 **	0.582 **	0.461 **	0.033	0.081	0.068	0.108 *	0.410 **	0.346 **
15. PA T-fw															-	0.395 **	0.561 **	0.670 **	0.044	−0.015	0.044	0.234 **	0.287 **	0.325 **
16. LPA EE-wy																-	0.351 **	0.662 **	−0.591 **	−0.384 **	−0.562 **	−0.250 **	−0.257 **	−0.312 **
17. LPA EE-wd																	-	0.843 **	−0.142 **	−0.515 **	−0.406 **	−0.009	−0.057	−0.045
18. LPA EE-fw																		-	−0.366 **	−0.532 **	−0.541 **	−0.135 **	−0.162 **	−0.185 **
19. MVPA EE-wy																			-	0.372 **	0.772 **	0.929 **	0.356 **	0.738 **
20. MVPA EE-wd																				-	0.848 **	0.270 **	0.885 **	0.766 **
21. MVPA EE-fw																					-	0.669 **	0.767 **	0.891 **
22. PA EE-wy																						-	0.309 **	0.742 **
23. PA EE-wd																							-	0.867 **
24. PA EE-fw																								-

**: Correlation significant at 0.01 level (bilateral); *: Correlation significant at 0.05 level (bilateral); Screen time (ST); Sleep Time (SlT); Light Physical Activity (LPA); Moderate–vigorous Physical Activity (MVPA); Physical Activity (PA); Time (T); Energy Expenditure (EE); Weekday (wy); Weekend (wd); Full Week (fw).

**Table 4:** Bivariate correlation between screen time, sleep time and physical activity for females.

	1	2	3	4	5	6	7	8	9	10	11	12	13	14	15	16	17	18	19	20	21	22	23	24
1. ST wy	-	0.552 **	0.778 **	−0.060	0.087	0.029	0.078	−0.013	0.019	−0.054	−0.108	−0.121 *	0.060	−0.087	−0.058	0.107	−0.023	0.037	−0.051	−0.107	−0.117 *	0.039	−0.129 *	−0.077
2. ST wd		-	0.954 **	−0.033	0.088	0.044	0.046	0.063	0.077	−0.038	−0.231 **	−0.190 **	0.033	−0.088	−0.039	0.048	−0.004	0.044	−0.046	−0.217 **	−0.179 **	−0.006	−0.231 **	−0.173 **
3. ST fw			-	−0.047	0.098	0.044	0.00.63	0.042	0.065	−0.048	−0.214 **	−0.187 **	0.047	−0.098	−0.051	0.075	−0.011	0.046	−0.053	−0.202 **	−0.177 **	−0.009	−0.221 **	−0.159 **
4. SlT wy				-	0.284 **	0.737 **	−0.884 **	−0.256 **	−0.480 **	0.026	−0.006	0.006	−1 **	−0.284 **	−0.537 **	−0.852 **	−0.245 **	−0.434 **	0.065	0.004	0.031	−0.664 **	−0.162 **	−0.426 **
5. SlT wd					-	0.858 **	−0.202 **	−0.796 **	−0.623 **	−0.099	−0.190 **	−0.180 **	−0.284 **	−1 **	−0.821 **	−0.201 **	−0.692 **	−0.607 **	−0.042	−0.161 **	−0.135 *	−0.216 **	−0.638 **	−0.570 **
6. SlT fw						-	−0.617 **	−0.699 **	−0.696 **	−0.056	−0.137 *	−0.124 *	−0.737 **	−0.858 **	−0.867 **	−0.598 **	−0.619 **	−0.660 **	0.005	−0.112 *	−0.079	−0.508 **	−0.537 **	−0.631 **
7. LPA T-wy							-	0.291 **	0.594 **	−0.489 **	−0.173 **	−0.338 **	0.884 **	0.202 **	0.445 **	0.949 **	0.270 **	0.534 **	−0.496 **	−0.174 **	−0.346 **	0.304 **	−0.001	0.140 *
8. LPA T-wd								-	0.827 **	−0.145 **	−0.443 **	−0.394 **	0.256 **	0.796 **	0.672 **	0.265 **	0.872 **	0.780 **	−0.190 **	−0.439 **	−0.406 **	0.032	0.128 *	0.109 *
9. LPA T-fw									-	−0.375 **	−0.420 **	−0.474 **	0.480 **	0.623 **	0.813 **	0.518 **	0.759 **	0.924 **	−0.409 **	−0.421 **	−0.489 **	0.024	0.070	0.063
10. MVPA T-wy										-	0.382 **	0.714 **	−0.026	0.099	0.050	−0.635 **	−0.440 **	−0.333 **	0.943 **	0.367 **	0.682 **	0.590 **	0.305 **	0.497 **
11. MVPA T-wd											-	0.905 **	0.006	0.190 **	0.126 *	−0.132 *	−0.390 **	−0.366 **	0.371 **	0.951 **	0.859 **	0.268 **	0.737 **	0.668 **
12. MVPA T-fw												-	−0.006	0.180 **	0.127 *	−0.292 **	−0.340 **	−0.415 **	0.682 **	0.867 **	0.952 **	0.450 **	0.682 **	0.711 **
13. PA T-wy													-	0.284 **	0.537 **	0.852 **	0.245 **	0.434 **	−0.065	−0.004	−0.031	0.664 **	0.162 **	0.426 **
14. PA T-wd														-	0.821 **	0.201 **	0.692 **	0.607 **	0.042	0.161 **	0.135 *	0.216 **	0.638 **	0.570 **
15. PA T-fw															-	0.391 **	0.630 **	0.767 **	−0.010	0.098	0.079	0.325 **	0.530 **	0.541 **
16. LPA EE-wy																-	0.278 **	0.529 **	−0.448 **	−0.131 *	−0.296 **	0.397 **	0.050	0.220 **
17. LPA EE-wd																	-	0.864 **	−0.164 **	−0.397 **	−0.359 **	0.070	0.259 **	0.223 **
18. LPA EE-fw																		-	−0.367 **	−0.375 **	−0.434 **	0.077	0.190 **	0.176 **
19. MVPA EE-wy																			-	0.395 **	0.730 **	0.642 **	0.305 **	0.521 **
20. MVPA EE-wd																				-	0.903 **	0.293 **	0.784 **	0.714 **
21. MVPA EE-fw																					-	0.495 **	0.707 **	0.750 **
22. PA EE-wy																						-	0.356 **	0.724 **
23. PA EE-wd																							-	0.902 **
24. PA EE-fw																								-

**: Correlation significant at 0.01 level (bilateral); *: Correlation significant at 0.05 level (bilateral); Screen time (ST); Sleep Time (SlT); Light Physical Activity (LPA); Moderate–vigorous Physical Activity (MVPA); Physical Activity (PA); Time (T); Energy Expenditure (EE); Weekday (wy); Weekend (wd); Full Week (fw).

## Data Availability

Not applicable.

## References

[1] Strain T., Brage S., Sharp S.J., Richards J., Tainio M., Ding D., Benichou J., Kelly P. (2020). Use of the prevented fraction for the population to determine deaths averted by existing prevalence of physical activity: A descriptive study. Lancet Glob. Health.

[2] Warburton D.E.R., Bredin S.S.D. (2017). Health benefits of physical activity: A systematic review of current systematic reviews. Curr. Opin. Cardiol..

[3] World Health Organization (2020). Guidelines on Physical Activity and Sedentary Behaviour.

[4] Ruiz I.M., Martín-Matillas M., Delgado-Fernández M., Delgado-Rico E., Campoy C., Verdejo-García A. (2021). Effect of increased physical activity on physical fitness in an overweight and/or obese group of adolescents. SPORT TK Rev. Euroam. Cienc. Deporte.

[5] Guthold R., Stevens G.A., Riley L.M., Bull F.C. (2020). Global trends in insufficient physical activity among adolescents: A pooled analysis of 298 population-based surveys with 1.6 million participants. Lancet Child Adolesc. Health.

[6] Lizandra J., Devís-Devís J., Valencia-Peris A., Tomás J.M., Peiró-Velert C. (2019). Screen time and moderate-to-vigorous physical activity changes and displacement in adolescence: A prospective cohort study. Eur. J. Sport Sci..

[7] Aibar A., Bois J.E., Zaragoza J., Generelo E., Paillard T., Fairclough S. (2014). Weekday and weekend physical activity patterns of French and Spanish adolescents. Eur. J. Sport Sci..

[8] Craggs C., van Sluijs E.M.F., Corder K., Panter J.R., Jones A.P., Griffin S.J. (2011). Do children’s individual correlates of physical activity differ by home setting?. Health Place.

[9] Xu F., Adams S.K., Cohen S.A., Earp J.E., Greaney M.L. (2019). Relationship between Physical Activity, Screen Time, and Sleep Quantity and Quality in US Adolescents Aged 16–19. Int. J. Environ. Res. Public Health.

[10] Farooq A., Martin A., Janssen X., Wilson M.G., Gibson A.M., Hughes A., Reilly J.J. (2020). Longitudinal changes in moderate-to-vigorous-intensity physical activity in children and adolescents: A systematic review and meta-analysis. Obes. Rev..

[11] Guimarães R.F., Mathieu M.E., Reid R.E.R., Henderson M., Barnett T.A. (2021). Physical Activity, Screen Time, and Sleep Trajectories From Childhood to Adolescence: The Influence of Sex and Body Weight Status. J. Phys. Act. Health.

[12] Groffik D., Fromel K., Badura P. (2020). Composition of weekly physical activity in adolescents by level of physical activity. BMC Public Health.

[13] Galindo-Perdomo F., Peiró-Velert C., Valencia-Peris A. (2021). Do Adolescents Who Meet Physical Activity Recommendations on Weekdays Also Meet Them on Weekends? A Cross-Sectional Study in Colombia. Int. J. Environ. Res. Public Health.

[14] Peiró-Velert C., Valenciano J., Beltrán-Carrillo V., Devís-Devís J. (2014). Variability of physical activity in 17–18 year-old Spanish adolescents by type of day and season. J. Sport Psychol..

[15] Peiró-Velert C., Devís-Devís J., Beltrán-Carrillo V.J., Fox K.R. (2008). Variability of Spanish adolescent’s physical activity patterns by seasonality, day of the week and demographic factors. Eur. J. Sport Sci..

[16] Dahlgren A., Sjöblom L., Eke H., Bonn S.E., Lagerros Y.T. (2021). Screen time and physical activity in children and adolescents aged 10–15 years. PLoS ONE.

[17] Hrafnkelsdottir S.M., Brychta R.J., Rognvaldsdottir V., Chen K.Y., Johannsson E., Gudmundsdottir S.L., Arngrimsson S.A. (2020). Less screen time and more physical activity is associated with more stable sleep patterns among Icelandic adolescents. Sleep Health.

[18] Gariepy G., Danna S., Gobiņa I., Rasmussen M., de Matos M.G., Tynjälä J., Janssen I., Kalman M., Villeruša A., Husarova D. (2020). How Are Adolescents Sleeping? Adolescent Sleep Patterns and Sociodemographic Differences in 24 European and North American Countries. J. Adolesc. Health.

[19] Kilius E., Samson D.R., Lew-Levy S., Sarma M.S., Patel U.A., Ouamba Y.R., Miegakanda V., Gettler L.T., Boyette A.H. (2021). Gender differences in BaYaka forager sleep-wake patterns in forest and village contexts. Sci. Rep..

[20] Jonasdottir S.S., Minor K., Lehmann S. (2021). Gender differences in nighttime sleep patterns and variability across the adult lifespan: A global-scale wearables study. Sleep.

[21] Grant V.M., Tomayko E.J., Kingfisher R.D. (2020). Sleep and Physical Activity Patterns in Urban American Indian Children. Am. J. Health Behav..

[22] Bejarano C.M., Carlson J.A., Conway T.L., Saelens B.E., Glanz K., Couch S.C., Cain K.L., Sallis J.F. (2021). Physical Activity, Sedentary Time, and Diet as Mediators of the Association Between TV Time and BMI in Youth. Am. J. Health Promot..

[23] Braig S., Genuneit J., Walter V., Brandt S., Wabitsch M., Goldbeck L., Brenner H., Rothenbacher D. (2018). Screen Time, Physical Activity and Self-Esteem in Children: The Ulm Birth Cohort Study. Int. J. Environ. Res. Public Health.

[24] O’Brien W., Issartel J., Belton S. (2018). Relationship between Physical Activity, Screen Time and Weight Status among Young Adolescents. Sports.

[25] Zhang Z.H., Li H.J., Slapsinskaite A., Zhang T., Zhang L., Gui C.Y. (2020). Accelerometer-measured physical activity and sedentary behavior in Chinese children and adolescents: A systematic review and meta-analysis. Public Health.

[26] Baddou I., El Hamdouchi A., El Harchaoui I., Benjeddou K., Saeid N., Elmzibri M., El Kari K., Aguenaou H. (2018). Objectively Measured Physical Activity and Sedentary Time among Children and Adolescents in Morocco: A Cross-Sectional Study. BioMed Res. Int..

[27] Costigan S.A., Barnett S., Plotnikoff R.C., Lubans D.R. (2013). The Health Indicators Associated with Screen-Based Sedentary Behavior Among Adolescent Girls: A Systematic Review. J. Adolesc. Health.

[28] Ghekiere A., Van Cauwenberg J., Vandendriessche A., Inchley J., Gaspar de Matos M., Borraccino A., Gobina I., Tynjälä J., Deforche B., De Clercq B. (2019). Trends in sleeping difficulties among European adolescents: Are these associated with physical inactivity and excessive screen time?. Int. J. Public Health.

[29] Hale L., Guan S. (2015). Screen time and sleep among school-aged children and adolescents: A systematic literature review. Sleep Med. Rev..

[30] Bandeira S.B., Ferreira-Lima W., Bandeira F., Bandeira F., Santos A., Molena C.A., Fuentes J.P. (2020). Sleep Hours: Risk behavior in adolescents from different countries. Cien Saude Colet..

[31] Tambalis K.D., Panagiotakos D.B., Psarra G., Sidossis L.S. (2018). Insufficient Sleep Duration Is Associated with Dietary Habits, Screen Time, and Obesity in Children. J. Clin. Sleep Med..

[32] Lee P.H., Tse A.C.Y., Wu C.S.T., Mak Y.W., Lee U. (2021). Temporal association between objectively measured smartphone usage, sleep quality and physical activity among Chinese adolescents and young adults. J. Sleep Res..

[33] Lang C., Kalak N., Brand S., Holsboer-Trachsler E., Puhse U., Gerber M. (2016). The relationship between physical activity and sleep from mid adolescence to early adulthood. A systematic review of methodological approaches and meta-analysis. Sleep Med. Rev..

[34] Gavela-Pérez T., Parra-Rodríguez A., Vales-Villamarín C., Pérez-Segura P., Mejorado-Molano F.J., Garcés C., Soriano-Guillén L. (2022). Relationship between eating habits, sleep patterns and physical activity and the degree of obesity in children and adolescents. Endocrinol. Diabetes Y Nutr..

[35] Mason J.O., Powell K.E. (1985). Physical activity, behavioral epidemiology, and public health. Public Health Rep..

[36] Dishman R.K., Heath G.W., Lee I. (2013). Physical Activity Epidemiology.

[37] Cale L. (1993). Monitoring Physical Activity in Children. Ph.D. Dissertation.

[38] Soler J.J., Generelo E., Zaragoza J., Julián J.A. (2010). Validity and Reliability Criteria for the “Four by One-Day Physical Activity Questionnaire” in Spanish Adolescents. Apunts. Phys. Educ. Sports.

[39] Beltrán-Carrillo V.J., Devís-Devís J., Peiró-Velert C. (2012). Physical activity and sedentary behaviour in adolescents from valencian region. Rev. Int. De Med. Y Cienc. Act. Física Y Deporte.

[40] Morales P. (2008). Statistics Applied to Social Sciences.

[41] Kjellenberg K., Ekblom Ö., Stålman C., Helgadóttir B., Nyberg G. (2021). Associations between Physical Activity Patterns, Screen Time and Cardiovascular Fitness Levels in Swedish Adolescents. Children.

[42] Moore J.B., Beets M.W., Morris S.F., Kolbe M.B. (2014). Day of the week is associated with meeting physical activity recommendations and engaging in excessive sedentary time in youth. J. Phys. Act. Health.

[43] Kallio J., Hakonen H., Syväoja H., Kulmala J., Kankaanpää A., Ekelund U., Tammelin T. (2020). Changes in physical activity and sedentary time during adolescence: Gender differences during weekdays and weekend days. Scand. J. Med. Sci. Sports.

[44] López A., Domínguez J., Portela I. (2020). Perceived barriers to physical exercise in adolescents: Differences according to gender, age and sport practice. J. Sport Psychol..

[45] Hernando A., Oliva A., Pertegal M.A. (2013). Gender differences in adolescents’ lifestyles. Psychosoc. Interv..

